# Anti-Metastatic Effects of Plant Sap-Derived Extracellular Vesicles in a 3D Microfluidic Cancer Metastasis Model

**DOI:** 10.3390/jfb11030049

**Published:** 2020-07-08

**Authors:** Kimin Kim, Jik-Han Jung, Hye Ju Yoo, Jae-Kyung Hyun, Ji-Ho Park, Dokyun Na, Ju Hun Yeon

**Affiliations:** 1 Department of Integrative Biosciences, University of Brain Education, Cheonan 31228, Korea; abc-632@hanmail.net (K.K.); hyeju_yoo@naver.com (H.J.Y.); 2 Department of Bio and Brain engineering, Korea Advanced Institute of Science and Technology, Daejeon 34141, Korea; jjhan@kaist.ac.kr (J.-H.J.); jihopark@kaist.ac.kr (J.-H.P.); 3 Electron Microscopy Research Center, Korea Basic Science Institute, Cheongju 28119, Korea; jaekyung.hyun@oist.jp; 4 School of Integrative Engineering, Chung-Ang University, Seoul 06911, Korea; blisszen@lile.cau.ac.kr

**Keywords:** cancer-associated fibroblasts, plant sap, extracellular vesicles, anti-metastatic effects, 3D microfluidics

## Abstract

Natural medicinal plants have attracted considerable research attention for their potential as effective drugs. The roots, leaves and stems of the plant, *Dendropanax morbifera*, which is endemic to southern regions of Asia, have long been used as a folk medicine to treat variety of diseases. However, the sap of this plant has not been widely studied and its bioactive properties have yet to be clearly elucidated. Here, we isolated extracellular vesicles from *D. morbifera* sap with the goal of improving the intracellular delivery efficiency and clinical effectiveness of bioactive compounds in *D. morbifera* sap. We further investigated the anti-metastatic effects of *D. morbifera* sap-derived extracellular vesicles (DMS-EVs) using a cancer metastasis model based on 3D microfluidic system that closely mimics the in vivo tumor environment. We found that DMS-EVs exerted a concentration-dependent suppressive effect on cancer-associated fibroblasts (CAFs), which are important mediators of cancer metastasis. DMS-EVs also altered expression level of genes, especially growth factor and extracellular matrix (ECM)-related genes, including integrin and collagen. Our findings suggest that DMS-EVs can act as anti-CAF agents to reduce CAFs in the tumor microenvironment. They further indicate the utility of our 3D microfluidic model for various drug-screening assays as a potential alternative to animal testing for use in validating therapeutic effects on cancer metastasis.

## 1. Introduction

Extracellular vesicles (EVs), which contain signal proteins, lipids, micro RNAs (miRNAs) and functional mRNAs, play an important role in intercellular communication in the extracellular space [1,2]. In addition to allowing cell-to-cell communication within the organism, EVs also allow cross-kingdom communication between plants and animals [3]. Recent research on edible plant-derived nanovesicles has concluded that miRNA-containing micro vesicles enable interspecies interactions between plants and mouse cells by transferring signals that affect the expression of genes and physiological regulation of the receiving cells [4]. Mu et al. have also reported that exosome-like nanoparticles isolated from edible plants induce activation of intestinal homeostasis, whereas Raimondo et al. isolated nano-sized vesicles from Citrus limon (lemon) and demonstrated their anticancer activities [5,6].

*Dendropanax morbifera* is a plant endemic to southern parts of Asia, a region with a rich folk medicine tradition and a history of commercial production of *D. morbifera* golden varnish. Recent studies have reported that extracts from roots, leaves and stems of *D. morbifera* exhibit antioxidant, anticancer and anti-diabetic effects, as well as anti-complement, anti-inflammatory and antiplasmodial activities [7,8,9,10,11,12,13]. However, whether EVs isolated from the sap of *D. morbifera* have anti-metastatic effects, especially effects on cancer-associated fibroblasts (CAFs), is not known.

Cancer metastasis is a manifestation of the acute invasive property of malignant cells, and is often associated with an unfavorable prognosis. The key factor governing the vigorousness of cancer metastasis is the formation and proliferation of CAFs. Tumor-stromal interactions contribute to the production of CAFs through endothelial–mesenchymal transition (EndMT). This process causes changes in normal cell morphology, promoting formation of filopodia in association with changes in the expression of genes that enhance motility [14]. The resulting sprouted and invading cells circulate within the body through the lymphatic system and blood vessels, and infiltrate into secondary metastatic sites. The three-dimensional (3D) cellular environment further contributes to tumor invasion across the extracellular matrix (ECM), reflecting the fact that interstitial flow forces CAFs to migrate in a specific direction [15,16]. ECM-related cellular enzymes, chemokines and other proteins also enhance cell motility by inducing cellular changes. Collectively, these observations suggests that CAFs play an essential role in tumor progression and growth into malignant tumors [17,18]. Therefore, CAF-targeted treatment has emerged as an area of great therapeutic interest for suppressing metastatic disease and preventing cancer metastasis [19].

In the current study, we tested the ability of *D. morbifera* sap-derived extracellular vesicles (DMS-EVs) to exert anti-metastatic effects through inhibition of CAF proliferation. To this end, we isolated EVs from *D. morbifera* sap and assessed differentiation of CAFs from monolayers of human umbilical vein endothelia cells (HUVECs) in a 3D microfluidic model incorporating ECM, interstitial flow, and melanoma-derived exosomes [14]. We analyzed changes in the phenotypic properties of CAFs and identified accompanying changes in gene expression induced by DMS-EV treatment that are associated with CAF suppression in our 3D microfluidics system. Our results indicate that DMS-EVs suppress cancer metastasis in a manner that depends on concentration and treatment frequency, and reflects changes in the expression of growth factor- and ECM-related genes, including integrin and collagen. We suggest that DMS-EVs are migrastatic candidates that interfere with the invasion and metastasis of cancer cells, and thus have the potential to be used as anticancer drugs. Our findings further suggest that our 3D microfluidics in vitro model of cancer metastasis could be used for various drug screening assays, especially in selection of new drug candidates from natural sources.

## 2. Materials and Methods

### 2.1. Isolation of DMS-EVs

We collected fresh sap from 20–30 years old *D. morbifera* trees between June and August in Bogil Island, located in Wando-gun, Jeollanam-do, South Korea with a warm and humid climate. We selected three trunks of 10 to 20 trees that naturally secrete the sap, scratched with knife, and carefully collected the exuded sap with spatula. EVs were from *D. morbifera* sap by mixing the sap with phosphate-buffered saline (PBS) at a ratio of 1:1 (*w*/*v*) and allowing the mixture to stand for 24–48 h. The supernatant was siphoned off with a syringe and filtered through a 0.22-μm filter membrane to remove large particles. The filtered sample was concentrated to less than 1 mL by centrifuging at 5000× *g* for 10 min in an Amicon Ultra-4 PL 100K centrifugal filter (Merck Millipore, Darmstadt, Germany). After centrifugation, the quantity of EVs was determined by measuring protein concentration using a Pierce BCA (bicinchoninic acid) protein assay kit (Thermo Scientific, Rockford, IL, USA).

### 2.2. Isolation of Cancerous Exosomes

B16BL6 murine melanoma cells (KCLB No. 8006; Korean Cell Line Bank [KCLB], Seoul, Korea) were cultured in minimum essential media alpha (α-MEM; Gibco), supplemented with 10% [*v*/*v*] fetal bovine serum (FBS; Rocky Mountain Biologicals, Missoula, MT, USA) and 1% [*v*/*v*] penicillin-streptomycin (Lonza, Basel, Switzerland). Before detachment of cells from the culture dish, the culture medium was replaced with Dulbecco’s Modified Eagle Medium (DMEM), supplemented with 10% exosome-depleted FBS and 1% [*v*/*v*] penicillin-streptomycin to remove interference from FBS exosomes in media. The culture medium was then collected and concentrated by tangential flow filtration using a membrane with a 100-kDa molecular weight cutoff (Sartorius, Goettingen, Germany). The resulting exosomes were filtered with a 0.22-μm filter and stored in a −80 °C freezer until use.

### 2.3. Collagen Gel Filling

5.0 mg/mL solution of collagen gel (pH 7.4) was prepared by add 10× phosphate buffered saline (PBS; Thermo Scientific, Waltham, MA, USA), 0.5N NaOH, distilled deionized water and collagen solution (8.9 mg/mL, rat tail collagen type I, high concentration; BD Bioscience, San Diego, CA, USA) in the given order. The hydrogel solution was then injected into the gel-filling region of microfluidic devices. Hydrogel-filled devices were place in a humidified, 37 °C incubator for 30 min. After incubation, all channels of the devices were filled with endothelial basal medium-2 (EBM-2; Lonza, Basel, Switzerland), and the devices were again placed in the incubator.

### 2.4. Cell Culture and Cell Seeding

HUVECs (CC-2517; Lonza, Basel, Switzerland) were cultured in endothelial growth medium (EGM-2; Lonza) at 37 °C in a 5% CO_2_ environment and used at fewer than 5 passages in all experiments. Approximately 50–60 μL of HUVEC suspensions (5 × 10^6^ cells/mL) was seeded into the central channel of microfluidic devices and allowed to attach. After attachment of HUVECs, all reservoirs of devices were filled with EGM-2 medium, and HUVECs were incubated for 2 days to allow formation of an endothelial monolayer.

### 2.5. CAFs Differentiation in 3D Microfluidic Device

HUVEC monolayers were reconstituted within 3D microfluidic devices supported by the collagen gel, which recapitulates a 3D tumor microenvironment. Cancer (melanoma)-derived exosomes (C-EVs) were delivered at a concentration of 50 µg/mL, after which interstitial fluid flow was provided through channels on one side through the collagen by virtue of height differences between side reservoirs. Our previous studies showed that the predicted interstitial flow rates in the device were range from 0.1–0.7 µm/s, which was pathophysiological level of interstitial flow [14,16]. CAFs differentiated and showed increased sprouting from the HUVEC monolayer towards the ECM, against the direction of interstitial fluid flow.

### 2.6. Uptake of DMS-EVs by CAFs

Cellular uptake of DMS-EVs was analyzed by fluorescence microscopy. DMS-EVs extracted as described above were tagged by incubating with red Di-I stain (1 µg/mL), a lipophilic dye. After DMS-EV treatment and uptake by HUVECs and CAFs in the 3D microfluidic device, the medium was removed and cells were fixed by incubating with 4% paraformaldehyde. The cells were prepared for staining by adding 0.15% Triton X-100 and incubating for 30 min at room temperature, after which all solutions were removed and wells were filled with 3% bovine serum albumin (BSA). After incubating for 1 h at room temperature, the solutions were again removed and Actin green 488 was added to stain the cytoplasm of the cells. Cells were then incubated for 30 min at room temperature, washed three times with 1% BSA, and incubated at room temperature for 15 min with Hoechst33342, which stains nuclei. The wells were then washed with 1% BSA and filled with PBS, after which cells were imaged under a fluorescence microscope (Leica Microsystems, Wetzlar, Germany).

### 2.7. LIVE/DEAD Viability Assay

The viability of mammalian cells was tested using a LIVE/DEAD viability/cytotoxicity kit (ThermoFisher Scientific, Rockford, IL, USA) as described by the manufacturer. Briefly, after culturing as described in the text, the medium was removed and cells were washed three times with PBS. Staining solution, prepared by mixing calcein AM and ethidium homodimer-1, was then added and cells were incubated for about 30 min at room temperature. The percentage of live cells stained by calcein with green fluorescence converted from calcein-AM by intracellular esterase activity and dead cells stained with orange/red by ethidium homodimer-1 (EthD-1) was assessed by imaging cells under a fluorescence microscope (Carl Zeiss, Jena, Germany).

### 2.8. Dynamic Light Scattering

Hydrodynamic size and zeta potential of DMS-EVs were determined using dynamic light scattering (DLS) and a Zetasizer (Malvern Instruments, Worcestershire, UK). Isolated EVs were placed in a thermostatic cell at 20 °C. The size distribution was determined by measuring hydrodynamic size. Thereafter, samples were mixed with distilled water at a ratio of 95:3 (*v*/*v*) for measurement of zeta potential. Diluted samples were inserted into the folded capillary cell (DTS1070; Malvern instrument) and detected using the same equipment indicated above.

### 2.9. Nanoparticle Tracking Analysis

The size distribution of particles in the sample was further determined by nanoparticle tracking analysis (NTA), which assesses the combined properties of light scattering and Brownian motion. Samples suspended in liquid were diluted and visualized with a Nanosight instrument (Malvern Instruments, Worcestershire, UK) at a temperature of 25 °C using a 488 nm laser.

### 2.10. Nanostring nCounter Assay

Nanostring nCounter assays (Nanostring Technologies, Inc., Seattle, WA, USA) were conducted according to the procedure outlined by the manufacturer. All tags were removed except for the DNA tags ligated onto the 3′-end of each miRNA in the samples. After each miRNA was uniquely represented, the samples were hybridized with tag-specific nCounter capture and barcoded reporter probes by reacting with 5 mL of 5×-diluted sample preparation reaction solution. Total RNA samples (100 ng) were incubated at 64 °C for at least 18 h. All excess capture and reported probes were removed. Target RNA molecules in the sample were quantified and classified by counting each fluorescent barcode with the nCounter digital analyzer.

### 2.11. Statistical Analysis

All data were obtained through three replicate experiments. Statistical analyses were performed using two-way analysis of variance (ANOVA) followed by Dunnett’s multiple comparisons test with GraphPad Prism (GraphPad Software Inc., La Jolla, CA, USA). *p*-value < 0.05 was considered statistically significant.

## 3. Results and Discussion

### 3.1. Characterization of DMS-EVs

**Figure 1:**
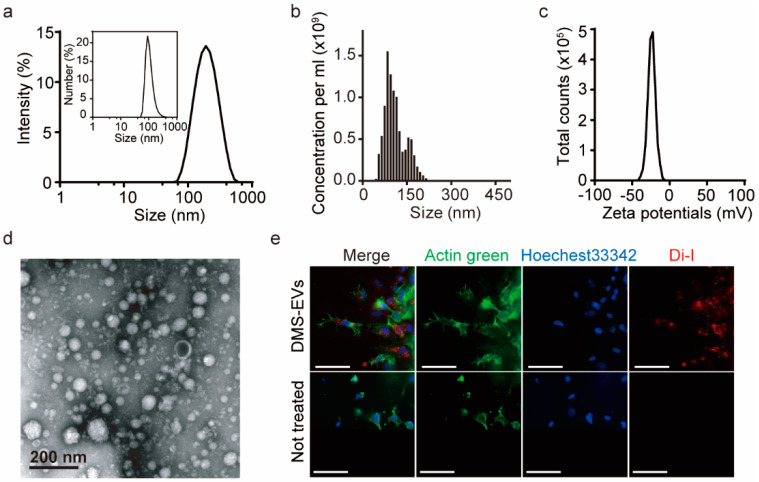
Characterization of extracellular vesicles (EVs) derived from *D. Morbifera* sap. (**a**) Size distribution of *Dendropanax morbifera* sap-derived extracellular vesicles (DMS-EVs), determined from intensity curves of dynamic light scattering (DLS) measurements. Inset: Number vs. size distribution; (**b**) Nanoparticle tracking analysis (NTA) measurements of the concentration of *D. morbifera* sap-derived extracellular vesicles (DMS-EVs); (**c**) Zeta potential distribution of *D. morbifera* sap-derived extracellular vesicles (DMS-EVs); (**d**) Transmission electron microscopy (TEM) images of DMS-EVs. Scale bar: 200 nm; (**e**) Representative fluorescence microscopic images showing cellular uptake of fluorescently labeled extracellular vesicles (EVs); Di-I (red), Actin green (green), Hoechest33342 (blue). Scale bar: 75 μm.

DMS-EVs were isolated using a sequential centrifugation step and filtration approach based on mammalian exosome isolation techniques, and their size distribution was evaluated by nanoparticle tracking analysis (NTA) and dynamic light scattering (DLS) analysis (Figure 1a,b). Both NTA and DLS analyses yielded estimates of size ranging from 100 to 200 nm, which is consistent with transmission electron microscopy (TEM) images, which showed nearly spherical vesicles (Figure 1d). DMS-EVs were similar to naturally occurring mammalian exosomes (30–200 nm). As shown in Figure 1b, the concentration of DMS-EVs was 1.02 × 10^9^ particles/mL, and particles displayed a negative zeta potential value of −25 mV. Zeta potential provides a measure of dispersion stability, a characteristic property of the population of exosomes that reflects interactions among charged particles. A higher zeta potential results in greater electrostatic mobility between particles and a greater tendency to resist aggregation. Vesicles with minimum zeta potentials of ±20 mV are moderately stable [20]. Thus, given their moderately negative charge (−25 mV), DMS-EVs exhibit mutual repulsion and are able to form a stable suspension (Figure 1c).

Fluorescent DMS-EVs, labeled with red Di-I and incubated with CAFs as described in Materials and Methods, were widely distributed within cells, including in the cytoplasm and around nuclei (Figure 1e), indicating that DMS-EVs are readily internalized by CAFs. To further evaluate the cellular uptake of DMS-EVs, we treated HUVECs with DMS-EVs at 1 and 10 µg/mL in 96-well plates for different time periods (0, 1, 3, 6, 9, and 12 h). Uptake of DMS-EVs by HUVECs was concentration-dependent and increased over time before reaching saturation (Figure S1).

### 3.2. DMS-EV Treatment Reduces the Number of CAFs

**Figure 2:**
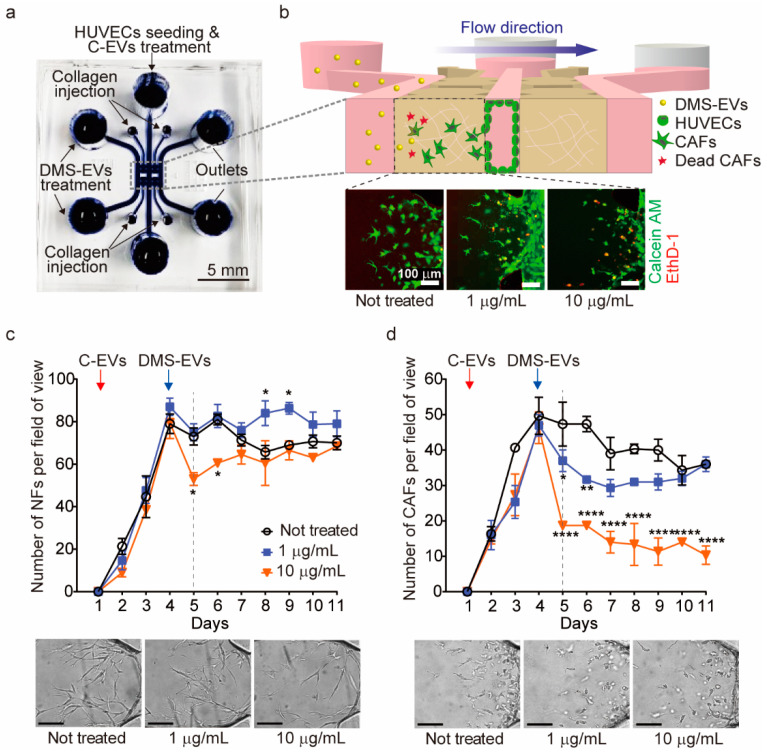
Comparison of *D. morbifera* sap-derived extracellular vesicles (DMS-EVs) effects on cancer-associated fibroblasts (CAFs) and normal fibroblasts (NFs). (**a**) A photograph of the 3D microfluidic chip for cancer metastasis model; (**b**) Schematic illustration showing the experimental setup for monitoring the reduction of cancer-associated fibroblasts (CAFs) by *D. morbifera* sap-derived extracellular vesicles (DMS-EVs) in a 3D microfluidic model, and live-dead images of cancer-associated fibroblasts (CAFs) following treatment with 1 or 10 μg/mL *D. morbifera* sap-derived extracellular vesicles (DMS-EVs); ethidium homodimer-1 (EthD-1) (red), Calcein AM (green); (**c**) Number of normal fibroblasts (NFs) over the entire culture period (10 days) after single-treatment with *D. morbifera* sap-derived extracellular vesicles (DMS-EVs), and bright field microscopy images showing the concentration-dependent reduction in normal fibroblasts (NFs) induced by *D. morbifera* sap-derived extracellular vesicles (DMS-EVs) treatment on day 5; (**d**) Number of cancer-associated fibroblasts (CAFs) over the entire culture period (10 days) after single-treatment with *D. morbifera* sap-derived extracellular vesicles (DMS-EVs), and bright field microscopy images showing the concentration-dependent reduction in cancer-associated fibroblasts (CAFs) induced by *D. morbifera* sap-derived extracellular vesicles (DMS-EVs) treatment on day 5. Scale bar: 100 μm. *, ** and **** indicate difference for *p* < 0.05, *p* < 0.01 and *p* < 0.0001 in comparison to cancer-associated fibroblasts (CAFs) with day 4, respectively (n = 3).

A 3D microfluidic model was used to monitor differentiation of CAFs from HUVECs (Figure 2a,b). To this end, we created a human endothelial monolayer by seeding HUVECs into the medium channel and filling other regions with type I collagen gel to create an ECM environment. Cancer (melanoma)-derived exosomes (C-EVs), which induce differentiation of endothelial cells into CAFs through EndMT [14], were introduced onto our in vitro model, and CAF movement towards the ECM, which is against the direction of interstitial fluid flow, was observed. CAFs were then treated with DMS-EVs in the direction of interstitial fluid flow and their survival rate was monitored in real time.

As a control, normal fibroblasts (NFs) were seeded into the medium channel, and inhibition rates for CAFs and NFs were determined in real time by counting the number of CAFs or NFs per field of view after single treatment with 1 or 10 µg/mL of DMS-EVs (Figure 2c,d). As shown in Figure 2c, treatment with 10 µg/mL of DMS-EVs caused an initial decrease (~30%) in the number of NFs, but these numbers recovered over time, approaching levels observed for NFs not treated with DMS-EVs; treatment with 1 μg/mL DMS-EVs had no effect on NF numbers. In contrast, treatment with either 1 or 10 μg/mL of DMS-EVs significantly inhibited the survival rate of CAFs, reducing the number of CAFs on day 5 by 30% and 60%, respectively, compared to that on day 4 when DMS-EVs was not treated.

Reprograming of fibroblasts into CAFs by cancer cells is mediated by miRNA. However, CAFs derived from tumor stroma have been reported to differ from normal stromal fibroblasts in terms of the expression of ECM proteins, pro-tumor factors, and cytokines [18,21]. Thus, the inhibitory effect of DMS-EVs on CAFs compared with NFs depends on the concentration of DMS-EVs. In our previous study, LC-MS/MS analysis was performed to investigate the protein composition of DMS-EVs, and we found that plant proteins of DMS-EVs were mainly associated with peroxidase [22]. Peroxidases are one of the plant defense proteins, known as antioxidant enzymes that detoxifie hydrogen peroxide. Recent studies have reported that secreted hydrogen peroxide by cancer cells triggers oxidative stress in adjacent cancer-associated fibroblasts [23]. Additionally, cancer-associated fibroblasts exhibit higher ROS level compared with normal fibroblasts [24]. The hydrogen peroxide leads to promote tumor growth, progression and metastasis. Therefore, peroxidase may contribute to new promising anticancer strategies.

**Figure 3:**
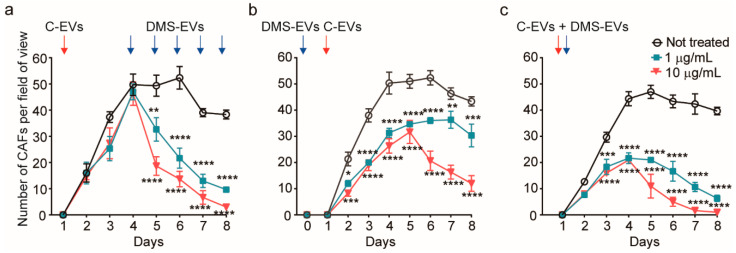
Quantitative assessment of cancer-associated fibroblasts (CAFs) viability in three different *D. morbifera* sap-derived extracellular vesicles (DMS-EVs)/cancer-derived EVs (C-EVs) treatment paradigms; (**a**) Number of cancer-associated fibroblasts (CAFs) over the entire culture period (10 days) after multiple, daily treatments with *D. morbifera* sap-derived extracellular vesicles (DMS-EVs). (**b**) Number of cancer-associated fibroblasts (CAFs) after pretreatment with cancer-derived EVs (C-EVs) and subsequent treatment with *D. morbifera* sap-derived extracellular vesicles (DMS-EVs); (**c**) Number of cancer-associated fibroblasts (CAFs) after co-treatment with *D. morbifera* sap-derived extracellular vesicles (DMS-EVs) and cancer-derived EVs (C-EVs). *, **, *** and **** indicate difference for *p* < 0.05, *p* < 0.01, *p* < 0.001 and *p* < 0.0001 in comparison to not treated (n = 3).

To further assess the effect of concentration and treatment procedure on the suppression of CAFs by DMS-EVs, we investigated three different conditions: repetitive treatment, pre-treatment before adding cancer-derived EVs (C-EVs) and co-treatment with C-EVs and DMS-EVs together (Figure 3).

For repetitive treatment, CAFs were treated with DMS-EVs every 24 h. With this experimental paradigm, after the initial DM-EV treatment, the inhibition rate and number of CAFs were similar regardless of the concentration of DM-EVs, and 4 days were required to completely suppress CAFs (Figure 3a). Thus, from the standpoint of therapeutic validation, both repeated and single DMS-EV treatments at 10 µg/mL, and repeated DMS-EV treatments at 1 µg/mL inhibited the growth of CAFs.

For pre-treatment, CAFs were treated with 1 or 10 µg/mL of DMS-EVs followed by treatment with C-EVs. In these experiments pre-treatment with 1 or 10 µg/mL of DMS-EVs decreased the survival of CAFs by 37% and 47%, respectively, on day 4 compared with DMS-EV–untreated CAFs. To date, there is limited evidence of the effectiveness of natural products in preventing cancer or cancer recurrence [25]. Thus, our demonstration that pre-treatment with DMS-EVs contributed to cancer prevention by further inhibiting C-EV–induced CAF differentiation from HUVECs is particularly noteworthy. Moreover, these findings suggest that our 3D microfluidics in vitro model of cancer metastasis could be used for various drug-screening assays, especially in the selection of natural products for cancer prevention.

In the co-treatment paradigm, DMS-EVs and C-EVs were applied together on a human endothelial monolayer our in vitro model. Co-treatment with 1 or 10 µg/mL of DMS-EVs together with C-EVs decreased CAF survival by 55% and 76%, respectively, on day 4 compared with DMS-EV–untreated CAFs. There is increasing evidence that natural products from dietary sources can make an important contribution to cancer development, and that diet may affect outcomes after cancer diagnosis [26,27]. Our results obtained with the 3D in vitro model suggest that it could be possible to harness such preventive effects in the therapeutic inhibition of tumors.

### 3.3. Significantly Differentially Expressed Genes in DMS-EV–Treated CAFs

**Figure 4:**
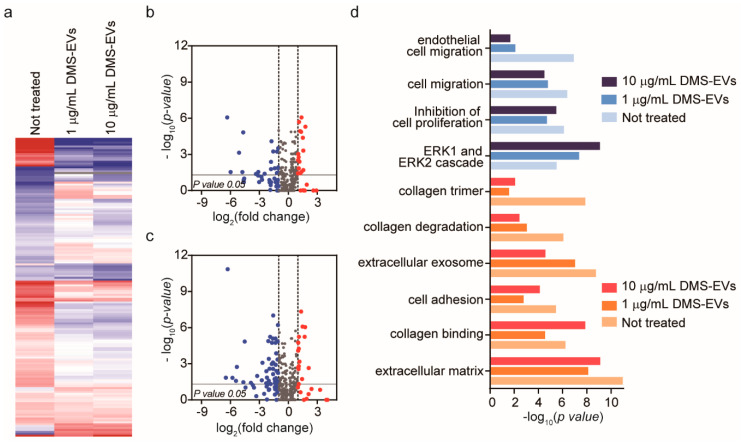
Significantly differentially expressed genes in cancer-associated fibroblasts (CAFs) following treatment with *D. morbifera* sap-derived extracellular vesicles (DMS-EVs). (**a**) Hierarchical clustering analysis based on Euclidean distance reflecting treatment of cancer-associated fibroblasts (CAFs) with 1 or 10 µg/mL of *D. morbifera* sap-derived extracellular vesicles (DMS-EVs). Column color code intensity reflects the magnitude of fold increase (red) and decrease (blue). Red, log_2_ fold change > 1; blue, log_2_ fold change < −1; (**b**,**c**) Volcano plot showing differences in gene expression in cancer-associated fibroblasts (CAFs) between 1 and 10 µg/mL *D. morbifera* sap-derived extracellular vesicle (DMS-EV) treatment; (**d**) GO term enrichment analysis of differentially expressed mRNAs in *D. morbifera* sap-derived extracellular vesicle (DMS-EV)–treated cancer-associated fibroblasts (CAFs) (*p*-value < 0.05; log_2_ fold change > 1 or <−1). Blue, cell migration-related genes; red, extracellular matrix (ECM)-related genes.

To identify the major CAF-associated genes that are affected by DMS-EV treatment, we profiled mRNA expression using the Nanostring nCounter platform, which includes a panel of 770 genes known to be involved in metastasis. To this end, CAFs were treated with 1 or 10 μg/mL of DMS-EVs and differences in gene expression compared with untreated CAFs were assessed. Of the 770 genes in the Nanostring nCounter analysis system panel, approximately 20% are CAF-related genes. Heat maps used to compare differentially expressed gene levels between untreated CAFs and CAFs treated with 1 or 10 µg/mL of DMS-EVs showed clusters based on similarities and dissimilarities among groups (Figure 4a). Interestingly, a hierarchical clustering analysis revealed clear differences in groups between untreated CAFs and CAFs treated with 1 or 10 µg/mL of DMS-EVs. Volcano plots showed that approximately 9% of genes were down-regulated and 5% of genes were up-regulated in CAFs treated with 1 µg/mL of DMS-EVs (Figure 4b), and about 15% of genes were down-regulated and 5% of genes were up-regulated in CAFs treated with 10 µg/mL of DMS-EVs (Figure 4c). Thus, the proportion of down-regulated genes in DMS-EV–treated CAFs compared with untreated CAFs increased in a manner that depended on the concentration of DMS-EVs. This dose-dependent decrease in CAF-related genes suggests that DMS-EVs are a promising therapeutic approach for the treatment of metastasis.

We then performed a Gene Ontology (GO) enrichment analysis of differently expressed mRNAs with critical functions in CAFs, comparing untreated CAFs with CAFs treated with 1 or 10 µg/mL of DMS-EVs, as shown in Figure 4d. The top 10 differently expressed mRNAs in CAFs were included in this GO enrichment analysis. We found that the most enriched GO terms were for growth factors involved in cell migration, including endothelial cell migration, and ECM genes related to integrin and collagen, including collagen trimers, collagen degradation, and extracellular exosomes. CAFs secrete growth factors, such as tumor growth factor-beta (TGF-β) and platelet-derived growth factor (PDGF), that stimulate tumorigenesis and migration of cancer cells, and are involved in tumor cell invasion by participating in EndMT, enhancing angiogenesis and mediating immune evasion of tumor cells [28,29]. CAFs also express ECM molecules (collagen) and contribute to remodeling via integrins, which are widely expressed ECM receptors that act through adhesion molecules to mediate interactions between the ECM and the actin cytoskeleton. Collectively, these results indicate that the expression of genes related to migration and ECM were among the most prominently changed genes in DMS-EV–treated CAFs compared with untreated CAFs.

### 3.4. DMS-EV-Induced Changes in Expression Levels of Migration-Related Genes in CAFs

**Figure 5:**
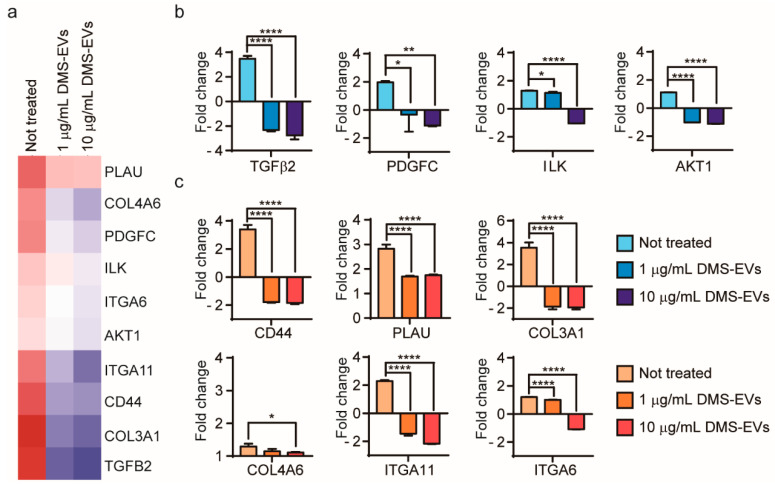
Comparative gene expression analysis in *D. morbifera* sap-derived extracellular vesicle (DMS-EV)–treated cancer-associated fibroblasts (CAFs). (**a**) Heat map comparing significantly differentially expressed genes between 1 and 10 µg/mL *D. morbifera* sap-derived extracellular vesicles (DMS-EVs) treatment in cancer-associated fibroblasts (CAFs); (**b**) Fold changes in expression levels of transforming growth factor beta 2 (*TFG-β2*), platelet-derived growth factor C (*PDGFC*), integrin-linked kinase (*ILK*), serine/threonine kinase 1 (*AKT1*), (**c**) Fold changes in expression levels of *CD44*, plasminogen activator (*PLAU*), collagen type III alpha 1 chain (*COL3A1*), collagen type IV alpha 6 chain (*COL4A6*), integrin α11 (*ITGA11*), and integrin α6 (*ITGA6*) genes. Blue, cell-migration related genes; red, extracellular matrix (ECM)-related genes. *, ** and **** indicate difference for *p* < 0.05, *p* < 0.01 and *p* < 0.0001 in comparison to not treated, respectively (n = 3).

To further investigate genes whose expression was changed in CAFs by DMS-EVs in a concentration-dependent manner, we focused on 10 genes that showed different degrees of differential expression between treatment with 1 and 10 µg/mL of DMS-EVs (Figure 5a): CD44, transforming growth factor beta 2 (TFG-β2), plasminogen activator (PLAU, or uPA), platelet-derived growth factor C (PDGFC), collagen type III alpha 1 chain (COL3A1), collagen type IV alpha 6 chain (COL4A6), integrin α6 (ITGA6), integrin α11 (ITGA11), integrin-linked kinase (ILK), and serine/threonine kinase 1 (AKT1).

TFG-β2, which was significantly upregulated in CAFs in the absence of DMS-EVs treatment, was decreased by 5.8 and 6.2-fold at 1 and 10 µg/mL of DMS-EVs, respectively, indicating a dominant effect of DMS-EVs on CAFs (Figure 5b). The TFG-β pathway is increasingly considered a therapeutic target, not only because of its role in malignization of the cancer ecosystem, but also because of its capacity to activate pro-tumorigenic differentiation of stromal cells. Inhibitors that block TFG-β signaling have been reported to have benefits for patients at risk of developing metastatic disease [30,31]. As shown in Figure 5b, expression of the PDGF gene, which was similarly upregulated in CAFs in the absence of treatment, was decreased by 2.3 and 3.1-fold by treatment with 1 and 10 µg/mL of DMS-EVs, respectively (Figure 5b). PDGF is a mediator of CAF-induced angiogenesis, and accelerates cancer cell proliferation through recruitment and activation of CAFs in malignant melanoma. Blocking PDGFC has been reported to reduce the growth and angiogenesis of tumors that are resistant to anti-VEGF therapies [32,33,34]. The ILK gene, a downstream target of TFG-β, was also reduced by DMS-EVs (Figure 5b); interestingly, studies have shown that silencing ILK inhibits cancer cells stimulated by TFG-β2 [35]. AKT1 was also downregulated by 1 and 10 µg/mL of DMS-EVs (Figure 5b). AKT signaling is another pathway that may have the potential to activate and induce cell proliferation, indicating that downregulation of AKT expression could inhibit tumor cell growth and promote apoptosis [36,37].

### 3.5. DMS-EV-Induced Changes in the Expression Levels of ECM-Related Genes in CAFs

During cancer progression, CAFs are directly involved in dysregulated collagen turnover, which leads to increased stiffening of the tissue [38,39]. The major components of the ECM are collagens, which constitute up to 30% of total ECM proteins. Type III collagen is a fibrillary-forming collagen that influences invasion and metastasis, whereas type IV collagen is major component of network-forming collagens comprising the basal membrane, where it serves an important molecular filtration function. We found that type III collagen was decreased by 5.4- and 5.5-fold by 1 and 10 µg/mL of DMS-EVs, respectively, which is the second-highest fold change in gene expression observed in this experiment; by comparison, the corresponding decreases in type IV collagen were much smaller (0.1 and 0.2-fold) (Figure 5c). Among integrin α isoforms, ITGA11 and ITGA6 mRNAs were decreased by DMS-EVs (Figure 5c). Integrin α11 expression in particular is a biomarker for activated CAFs and is associated with increased ECM stiffness; thus, these findings suggest the involvement of ECM reorganization as an underlying mechanism in the resulting enhanced cancer cell proliferation and migration. Because reduced collagen cross-linking leads to repression of tumor progression and knockout of the ITGA11 gene results in diminished migration and invasion of tumor cells [40,41,42], integrin α is considered a stromal therapeutic target. CD44, a cell surface adhesion receptor that mediates cell–ECM interactions, can activate various signaling pathways that lead to cell proliferation, migration, and invasion. Studies have shown that knocking out CD44 suppresses the EMT phenotype (Figure 5c) [35].

Although these findings reveal changes in CAF-associated genes with DMS-EV treatment, whether DMS-EVs contribute directly to the inhibition of genes associated with the reduction in the number CAFs will require further investigation, because these genes exhibit interactions among the various ECM-related pathways. The traditional therapeutic modalities against cancer are chemotherapy, radiation therapy, and antiangiogenic therapy [19]. However, CAFs-related metastasis—and chemoresistance—is supported by the microenvironment. These results suggest that DMS-EVs are effective for targeted stromal therapy, which may make an important contribution to clinical outcome in patients with metastatic malignancy. Naturally derived plant components have been shown to prevent the initiation and progression of cancer cells and suppress the cancer microenvironment, but because of their poor absorption and weak resorption, they are not always effective [43]. Thus, an integrated nano-medicine approach using natural products-based nanotechnology serves as a platform for the development of novel antimetastatic agents. As nano-sized, naturally occurring, plant-derived extracellular vesicles, DMS-EVs may provide a new direction for future improvements in cancer treatment.

## 4. Conclusions

We investigated the inhibitory effects of DMS-EVs against CAFs in a 3D microfluidic model. We quantitatively analyzed the concentration-dependent effects of DMS-EVs on the phenotypic properties and viability of CAFs in real time, and performed a genotypic analysis to identify and determine the function of the dominant genes associated with CAFs that were affected by DMS-EVs treatment. Results obtained using our 3D in vitro model suggest that naturally derived nano-vesicles from plants have the potential to exert concentration- and time-dependent preventive and suppressive effects on the tumor microenvironment. Genes that showed differential expression in response to treatment with DMS-EVs preferentially included growth factors and ECM-related proteins, including integrins and collagens. On the basis of our results, we propose that an integrated nano-medicine approach using natural plant-based nanotechnology may provide a new direction for improving targeted stromal therapy. Further in vivo pre-clinical research and clinical studies are needed to develop safer and more effective anticancer metastasis treatments in the future.

## Supplementary Materials

The following are available online at https://www.mdpi.com/2079-4983/11/3/49/s1. Figure S1: Uptake of DM-EVs into HUVECs.
