# Supplementary material for: Anti-Metastatic Effects of Plant Sap-Derived Extracellular Vesicles in a 3D Microfluidic Cancer Metastasis Model
Source: J Funct Biomater. 2020 Jul 8;11(3):49. doi: 10.3390/jfb11030049 (PMC7563847; doi:10.3390/jfb11030049)
Supplement: Supplementary file 1 [file jfb-11-00049-s001.pdf]

Journal of Functional Biomaterials

Supplementary

Anti-Metastatic Effects of Plant Sap-Derived Extracellular Vesicles in a 3D Microfluidic Cancer Metastasis Model

Kimin Kim [1], Jik-Han Jung [2], Hye Ju Yoo [1], Jae-Kyung Hyun [3], Ji-Ho Park [2], Dokyun Na [4] and Ju Hun Yeon [1,*]

[1] Department of Integrative Biosciences, University of Brain Education, Cheonan 31228, Korea; abc-632@hanmail.net (K.K.); hyeju_yoo@naver.com (H.J.Y.)
[2] Department of Bio and Brain engineering, Korea Advanced Institute of Science and Technology, Daejeon 34141, Korea; jjhan@kaist.ac.kr (J.-H.J.); jihopark@kaist.ac.kr (J.-H.P.)
[3] Electron Microscopy Research Center, Korea Basic Science Institute, Cheongju 28119, Korea; jaekyung.hyun@oist.jp
[4] School of Integrative Engineering, Chung-Ang University, Seoul 06911, Korea; blisszen@lile.cau.ac.kr
* Correspondence: jhyeon@ube.ac.kr; Tel.: +82-41-529-2621; Fax: +82-41-529-2674

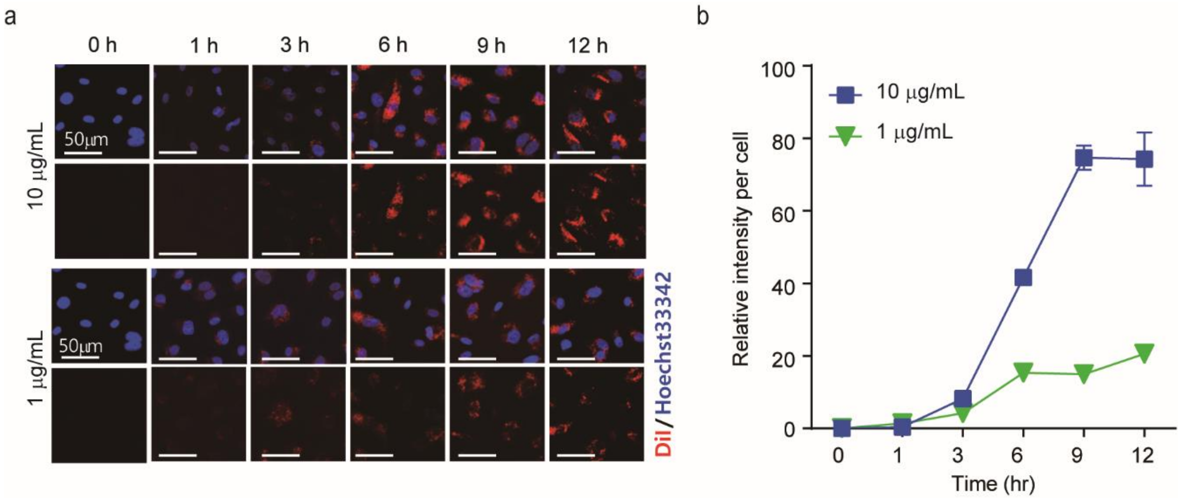

Figure S1. Uptake of DM-EVs into HUVECs. (**a**) Representative fluorescence microscopic images of cellular uptake of fluorescently labeled DM-EVs. (**b**) A comparison of fluorescence intensity with different time of 0, 1, 3, 6, 9, 12 h, respectively. Scale bar: 50 μm.

© 2020 by the authors. Submitted for possible open access publication under the terms and conditions of the Creative Commons Attribution (CC BY) license (http://creativecommons.org/licenses/by/4.0/).
